# Simplification to atazanavir/ritonavir+lamivudine in virologically suppressed HIV-infected patients: 24-weeks interim analysis from ATLAS-M trial

**DOI:** 10.7448/IAS.17.4.19808

**Published:** 2014-11-02

**Authors:** Massimiliano Fabbiani, Simona Di Giambenedetto, Eugenia Quiros-Roldan, Alessandra Latini, Vincenzo Vullo, Andrea Antinori, Antonella Castagna, Giancarlo Orofino, Daniela Francisci, Elisabetta Grilli, Giordanu Madeddu, Pierfrancesco Grima, Stefano Rusconi, Barbara Del Pin, Annalisa Mondi, Alberto Borghetti, Emanuele Focà, Manuela Colafigli, Andrea De Luca, Roberto Cauda

**Affiliations:** 1 Institute of Clinical Infectious Diseases, Catholic University of Sacred Heart, Rome, Italy; 2 University Division of Infectious and Tropical Dis, University of Brescia, Brescia, Italy; 3 Infectious Dermatology and Allergology Unit, IFO S. Gallicano Institute (IRCCS), Rome, Italy; 4 Department of Infectious Diseases, “La Sapienza” University, Rome, Italy; 5 Department of Infectious Diseases, National Institute for Infectious Diseases “Lazzaro Spallanzani” IRCCS, Rome, Italy; 6 Department of Infectious and Tropical Diseases, San Raffaele Scientific Institute, Milan, Italy; 7 Infectious and Tropical Diseases Unit, Amedeo di Savoia Hospital, Turin, Italy; 8 Infectious Diseases Clinic, University of Perugia, Perugia, Italy; 9 Systemic Infections and Immunodeficiency Unit, National Institute for Infectious Diseases “Lazzaro Spallanzani” IRCCS, Rome, Italy; 10 Department of Clinical and Experimental Medicine, University of Sassari, Sassari, Italy; 11 Division of Infectious Diseases, “S. Caterina Novella” Hospital, Galatina, Italy; 12 Infectious Disease Unit, University of Milan, Milan, Italy; 13 Unit of Infectious Diseases, S.M. Annunziata Hospital, Florence, Italy; 14 Infectious Diseases Unit, Siena University Hospital, Siena, Italy

## Abstract

**Introduction:**

We report interim 24-weeks efficacy data of ATLAS-M trial, a phase IV, multicentre, open-label, randomized study designed to show 48-weeks, non-inferior efficacy (margin of −12%) of treatment simplification to atazanavir/ritonavir (ATV/r)+lamivudine (3TC) versus maintaining 3-drugs ATV/r-based cART.

**Methods:**

Subjects on ATV/r+2 NRTIs, without previous treatment failure (TF), with HIV-RNA <50copies/mL for >3 months and CD4>200 cells/mm^3^ for >6 months were eligible. At baseline, patients were randomized to switch to ATV/r+3TC (arm one) or to maintain the original 3-drug regimen (arm two). Primary endpoint: proportion of patients free of TF at week 48. TF was defined as treatment modification for any reason, including virological failure (VF=two consecutive HIV-RNA>50 copies/mL or a single value >1000 copies/mL). Enrollment of 266 patients was planned.

**Results:**

A total of 266 patients (78% males, median age 44 years, median CD4 603 cells/µL, 79% treated with a tenofovir-containing backbone) were enrolled. At the time of analysis, 24 weeks data were available for 84 and 87 patients in arm one and two, respectively. At baseline, subjects in the two arms did not differ for the main characteristics. At 24 weeks, at the intention to treat analysis the proportion of patients free of TF was 91.7% (95% CI 85.8–97.6) and 85.1% (95% CI 77.6–92.6) in arm one and two, respectively (difference +6.6%, 95% CI −2.9/+16.1). VF was observed in two patients randomized to arm one (one at baseline, before treatment simplification) and one to arm two without resistance mutations. Clinical and laboratory adverse events occurred at similar rates in the two arms. At week 24, patients in arm one showed a greater increase in CD4 (mean change +90 vs +10 cells/µL, p=0.007). A greater increase in total cholesterol (+18 vs −2 mg/dL, p<0.001), HDL (+4 vs +0 mg/dL, p=0.001) and LDL (+12 vs +0 mg/dL, p=0.001) was also observed in arm one without differences in other lipid parameters. Renal function showed a significant improvement in arm one (mean change in eGFR +5 vs −2 mL/min/1.73m^2^ in arm two, p=0.001). No significant differences in bilirubin levels or other laboratory parameters were observed between the two arms.

**Conclusions:**

This interim analysis suggests a 24-weeks non-inferior efficacy of treatment simplification to ATV/rit+3TC as compared to continuation of ATV/rit +2 NRTI in virologically suppressed patients. Follow-up until 48-weeks is scheduled to confirm these data.

